# Retrospective Evaluation of Omalizumab Treatment Efficacy in Patients with Bullous Pemphigoid

**DOI:** 10.3390/jcm14186382

**Published:** 2025-09-10

**Authors:** Nazlı Caf, Zafer Türkoğlu, Göknur Özaydın Yavuz, İrem Doğan, Sümeyye Nur Aydın, İkram Kevser Atilla, Hafize Uzun

**Affiliations:** 1 Department of Dermatology and Venerology, Faculty of Medicine, Istanbul Atlas University, 34403 Istanbul, Türkiye; 2 Department of Dermatology and Venerology, Başakşehir Çam and Sakura City Hospital, Health Sciences University, 34480 Istanbul, Türkiye; 3 Department of Public Health, Istanbul Provincial Health Directorate, 34122 Istanbul, Türkiye; 4 Department of Medical Biochemistry, Faculty of Medicine, Istanbul Atlas University, 34403 Istanbul, Türkiye

**Keywords:** BPDAI score, bullous pemphigoid, corticosteroids, IgE, omalizumab, pruritus VAS score

## Abstract

**Background/Objectives**: Bullous pemphigoid (BP) is a manageable condition, and the primary goal of treatment is to control the disease while minimizing the use of corticosteroids due to their potential side effects with long-term use. The primary aim of this study was to assess the effectiveness of omalizumab (OMZ) treatment in bullous pemphigoid patients using both objective and subjective indicators, including bullous pemphigoid disease area index (BPDAI) score, peripheral eosinophil count, serum total IgE level, systemic corticosteroid dosage, and pruritus severity (VAS pruritus). The secondary aim was to explore potential predictors of treatment response, such as baseline BPDAI, age, gender, lesion distribution, serum total IgE, peripheral eosinophil count, maximum and minimum corticosteroid dose, and comorbidities, as well as to evaluate the time to clinical response and corticosteroid tapering. **Methods**: This retrospective analysis included 25 BP patients treated with OMZ as add-on therapy to systemic corticosteroids between January 2023 and December 2024 at Health Sciences University, Başakşehir Çam and Sakura Training and Research Hospital, Dermatology and Venerology Clinic. No other systemic immunosuppressants were permitted. All patients were already receiving systemic corticosteroids at enrolment. This retrospective analysis included 25 BP patients receiving omalizumab (300 mg/4 weeks) as an add-on to systemic corticosteroids, initiated primarily for steroid-refractory disease and/or persistent, sleep-disrupting pruritus. Baseline was defined immediately before the first OMZ dose; assessments were performed at baseline and week 12. Clinical (BPDAI, VAS pruritus) and laboratory (eosinophil count, total IgE levels) parameters were assessed at baseline and week 12. **Results:** OMZ treatment significantly reduced disease severity, as evidenced by a mean decrease in the BPDAI score of 105.0 ± 48.9 (95% CI 84.8–125.2) compared to baseline (*p* < 0.001). Peripheral eosinophil count also decreased by 0.6 ± 0.3 (95% CI 0.4–0.7) after treatment (*p* < 0.001). Total serum IgE levels declined significantly in 92% of patients (95% CI 244.5–2171.3) compared to pretreatment (*p* < 0.001), although two patients (8%) showed an increase (202.0 ± 258.8) after OMZ treatment. OMZ treatment led to a mean systemic corticosteroid dose reduction of 37.0 ± 14.1 mg (95% CI 31.1–42.8 mg), with a median corticosteroid tapering time of 4 weeks (3.0–4.0). Additionally, pruritus severity, measured by pruritus VAS, decreased by 6.2 ± 1.4 (95% CI 5.6–6.7) following treatment (*p* < 0.001). OMZ was well tolerated, with no serious adverse events. **Conclusions**: Within a 12-week observation window, we observed improvements in disease activity and pruritus alongside reduced corticosteroid exposure. Given the retrospective, uncontrolled add-on design, these findings do not establish causality but support further prospective controlled evaluation of omalizumab as a steroid-sparing option. Importantly, OMZ treatment significantly reduced the mean corticosteroid dose, pruritus VAS score, total IgE levels, and eosinophil count, indicating therapeutic activity and supporting its use as an effective steroid-sparing option in the management of bullous pemphigoid.

## 1. Introduction

Bullous pemphigoid (BP) is the most common autoimmune blistering disease in the elderly, characterized by tense subepidermal blisters. In BP, the autoantibodies are directed against BP180 and BP230, which are components of hemidesmosomes [1]. BP is characterized by erythematous urticarial papule plaques and/or tense bullae on the skin. Apart from the skin, the oral mucosa and/or genital mucosa may also be affected, and the quality of life of patients may be greatly affected due to intense itching [2]. Systemic corticosteroids are still the main treatment approach in severe BP, but their long-term use can lead to serious side effects such as diabetes mellitus, hypertension, and/or osteoporosis. Other immunosuppressive agents also have a broad side effect profile and pose a risk to this already fragile group of patients [3]. Therefore, it is obvious that safer alternative treatments are needed for the management of this disease.

Omalizumab (OMZ) is a recombinant humanized monoclonal anti-IgE antibody, primarily approved for conditions such as chronic spontaneous urticaria and asthma [4]. However, in recent years, the role of IgE in the pathogenesis of BP has become better understood, leading to the consideration of omalizumab treatment in this BP patient population [1]. In recent years, positive treatment results have been obtained in various case series with OMZ in BP [5,6,7,8].

Studies on the effects of OMZ in patients with BP are limited, and large-scale cohorts are still lacking, which prevents a clear identification of predictors of treatment response. However, it is potentially effective, particularly in patients with elevated total IgE levels. OMZ has also been reported to yield promising results in patients who are refractory to, have contraindications to, and/or unresponsive to other treatments [5,6,7].

This study aimed to evaluate the efficacy of OMZ in managing BP, specifically assessing its impact on Bullous Pemphigoid Disease Area Index (BPDAI) scores, systemic corticosteroid dose reduction, pruritus–Visual Analog Scale (VAS) score, total IgE levels, and blood eosinophil count. The study also aimed to explore correlations between treatment response and factors such as baseline BP-disease severity, systemic corticosteroid use, and patient age, while also evaluating the potential of OMZ to reduce corticosteroid dependence and improve overall disease management.

## 2. Materials and Methods

### 2.1. Ethical Approval

The protocol for sample collection was approved by the University of Health Sciences, Başakşehir Çam and Sakura Education and Research Hospital, Clinical Research Ethics Committee (approval number: E-96317027-514.10-267063174; date: 6 February 2025). The study was performed according to the Helsinki Declaration.

### 2.2. Study Design and Population

This study retrospectively analyzed 25 patients with BP who were managed with omalizumab as an add-on to systemic corticosteroids between January 2023 and December 2024 at dermatology autoimmune bullous diseases outpatient clinic of Başakşehir Çam and Sakura Education and Research Hospital, a recognized tertiary care center.

BP predominantly affects the elderly population, and its incidence markedly increases after the age of 60. Therefore, we restricted our inclusion criteria to patients aged over 50 to reflect the typical epidemiological characteristics of the disease and ensure population homogeneity. This criterion also aligns with previous large-scale epidemiological studies reporting that BP is extremely rare in individuals under 50 years of age [9].

### 2.3. Inclusion Criteria

Patients over the age of 50 were included if they had complete clinical and demographic data; a confirmed diagnosis of BP based on histopathology, direct immunofluorescence, and/or ELISA detecting BP180 and BP230 antibodies; available peripheral blood eosinophil counts and total IgE levels; and documented BPDAI (bullous pemphigoid disease area index) and pruritus VAS scores at treatment initiation and the twelve-week follow-up. Only those who received omalizumab as adjunct therapy and had complete medical records were enrolled.

### 2.4. Exclusion Criteria

Patients under the age of 50 and those with missing parameters in their clinical files and medical records were excluded from the study. Patients receiving any systemic immunosuppressive/immunomodulatory drugs other than systemic corticosteroids at any time during treatment were excluded from the study.

### 2.5. Concomitant Treatments

At the time of omalizumab (OMZ) initiation, none of the patients were receiving other systemic immunosuppressive or immunomodulatory agents (e.g., azathioprine, mycophenolate mofetil, methotrexate, cyclophosphamide, dapsone, tetracyclines). All patients were already on systemic corticosteroids (CSs) at enrolment, in line with standard care for moderate–severe BP (typically 0.5–1.0 mg/kg/day prednisolone-equivalent). OMZ was subsequently initiated as an add-on within a steroid-sparing strategy owing to inadequate disease control (steroid-refractory) and/or CS-related adverse events/intolerance and to address severe pruritus, which may markedly affect quality of life. Baseline was defined as the visit immediately prior to the first OMZ dose. The choice of OMZ over other systemic immunosuppressants was guided by patient-specific factors: (1) advanced age with associated frailty; (2) formal contraindications or physician concerns regarding potential toxicity of alternative immunosuppressants (e.g., azathioprine, mycophenolate mofetil, methotrexate, cyclophosphamide); (3) at baseline, all patients had elevated total IgE levels; and (4) pruritus being the predominant and most burdensome symptom. Collectively, these considerations favored OMZ as a corticosteroid-sparing option with an acceptable safety profile in this cohort.

### 2.6. OMZ Dosing, Corticosteroid Therapy, Data Collection, and Clinical Response Assessment

All patients received OMZ at a dose of 300 mg subcutaneously every four weeks. The initial dose of systemic corticosteroid therapy before omalizumab was 0.5–1.0 mg/kg/day of oral prednisolone or equivalent. OMZ was added to the treatment regimen due to corticosteroid-refractory disease, related adverse events, and/or resistant pruritus. *Corticosteroids were tapered only after the pre-specified clinical improvement criteria were met, generally in ~4-week steps. This relatively early and stepwise reduction strategy was driven not only by disease control but also by patient-specific factors, including advanced age, comorbidities, intolerance, adverse effects to systemic corticosteroids, and/or persistent itch.*

Outcome time-points were baseline (immediately before the first OMZ injection) and week 12. Disease activity and symptom burden were assessed using the BPDAI (0–360; higher scores indicate more extensive activity) and pruritus VAS (0–10; 0 = no itch, 10 = unbearable itch). Time-to-onset of response was defined as the interval from the first OMZ injection to the first documentation of clinically significant improvement in pruritus VAS (≥3-point reduction) and/or BPDAI (≥20% reduction). Laboratory variables (peripheral eosinophil count and total IgE) were measured at the same time-points to explore associations with treatment response.

The pruritus VAS was scored on a 0–10 scale (0: no itching; 10: unbearable itching). BPDAI ranged from 0 to 360, with higher scores indicating more extensive disease activity.

The following data were systematically collected from electronic medical records and patients’ hardcopy follow-up files: age, sex, disease duration, comorbidities, prior treatments, omalizumab dosage and duration, baseline and 12-week eosinophil counts, total IgE levels, BPDAI scores, pruritus VAS scores, and any adverse effects. These variables were used to evaluate treatment efficacy and identify potential predictors of response.

Adverse effects during OMZ treatment were systematically monitored and recorded. In this cohort, OMZ was generally well tolerated, with no serious treatment-related adverse events observed. Minor side effects, if any, were transient and did not necessitate discontinuation of therapy.

### 2.7. Clinical Improvement Criteria

In routine care, an initial corticosteroid taper was allowed at clinician discretion once early improvement in disease activity and pruritus was observed. For statistical analyses, “response” was pre-specified as a ≥20% reduction in BPDAI and/or a ≥3-point decrease in pruritus VAS from the pre-OMZ baseline to week 12. Relapse was defined as the occurrence of new urticarial plaques, new blisters, or worsening pruritus requiring treatment escalation after initial improvement.

### 2.8. Laboratory Parameters

Blood samples were taken into standardized tubes without anticoagulants and containing dipotassium ethylenediaminetetraacetic acid (EDTA) for complete blood count (CBC) parameters. The CBC result was recorded with an automatic hematology analyzer (Sysmex XN-1000, Norderstedt, Germany).

Total IgE in peripheral blood was measured using an automated analyzer (COBAS 8000, ROCHE-2007, Tokyo, Japan). Serum levels of BP180 and BP230 autoantibodies were measured using a commercially available ELISA kit. Serum total IgE was measured in IU/mL, and peripheral eosinophil count was expressed as the absolute number of cells per microliter (cells/μL) (elevated total IgE was defined as a value above the laboratory-specific reference (normal) range).

### 2.9. Statistical Analysis

Statistical analyses were performed using SPSS v.26 (IBM Corp., Armonk, NY, USA) software. The conformity of the variables to a normal distribution was analyzed using the Shapiro–Wilk test, Q-Q plot, and histogram. As a result of the analysis, normally distributed variables were shown as mean ± standard deviation, and non-normally distributed variables were shown as median (25th–75th percentile). Categorical variables were expressed as frequency (percentage). In the evaluation of two independent groups, the Mann–Whitney U test was used when the distribution was not normal, and Student’s *t*-test was used when the distribution was normal. The Wilcoxon signed-rank test was used to perform two dependent group comparisons in continuous data. The relationship between the continuous variables was analyzed using the Pearson correlation test. In all analyses, *p* < 0.05 was considered significant.

## 3. Results

The mean age of the patients was 71.3 ± 8.4 years, and the mean disease duration was 4.4 ± 2.5 months; 76% of the patients were female. The most frequently involved areas were the trunk (96%), and extremities (92%), respectively. The mean time to onset of response to OMZ treatment was 6.4 ± 3.3 weeks. The median time to the first corticosteroid dose reduction was 4.0 weeks (IQR 3.0–4.0). Sociodemographic and clinical characteristics of the patients are presented in Table 1.

**Figure 1:**
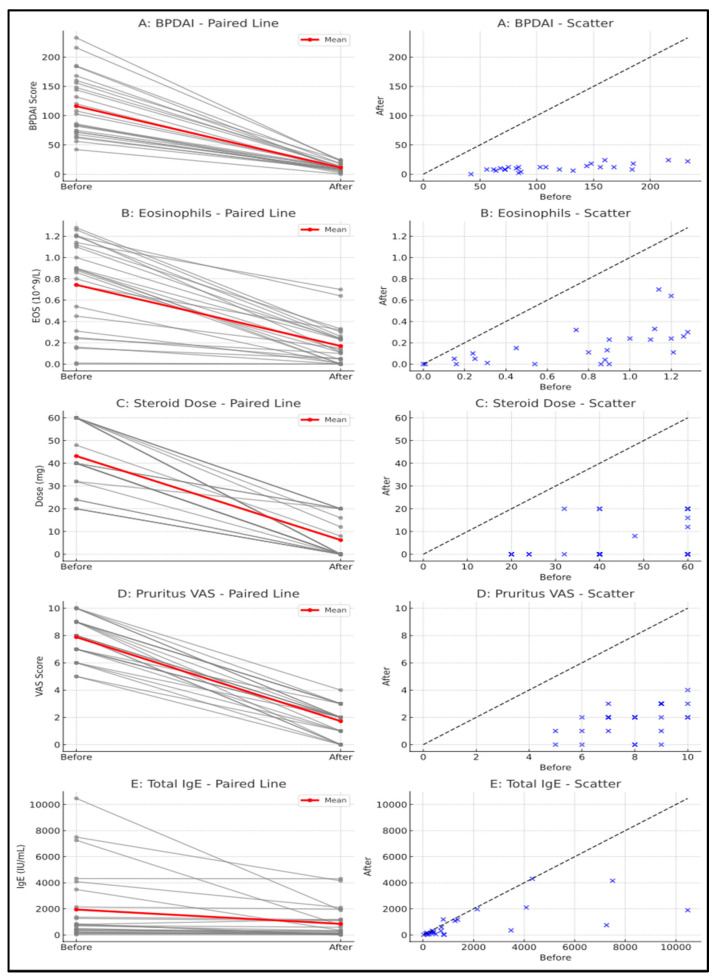
(**A**) Bullous pemphigoid disease area index (BPDAI) score change during omalizumab (OMZ) treatment; (**B**) eosinophil levels change during OMZ treatment; (**C**) decline in systemic corticosteroid dosage after OMZ treatment; (**D**) pruritus–visual analogue scale (VAS) score changes during OMZ treatment; (**E**) decline in serum total IgE levels during OMZ treatment.

OMZ caused a mean reduction of 105.0 ± 48.9 (95% CI 84.8–125.2) in the BPDAI score compared to the pretreatment period (*p* < 0.001). Eosinophil count was reduced by 0.6 ± 0.3 (95% CI 0.4–0.7) after treatment (*p* < 0.001). OMZ treatment resulted in a mean corticosteroid dose reduction of 37.0 ± 14.1 mg (95% CI 31.1–42.8 mg) from baseline (*p* < 0.001). By week 12, the median systemic corticosteroid dose was 0 mg (IQR 0–18 mg), indicating substantial tapering at follow-up. Patients’ pruritus level was evaluated with pruritus VAS score, and pruritus VAS score decreased by 6.2 ± 1.4 (95% CI 5.6–6.7) after treatment (*p* < 0.001). After treatment, five (20%) patients had a pruritus VAS score of 0. *At baseline, 25/25 patients had high total IgE. By week 12, total IgE declined relative to baseline in 23/25 (92%), whereas 2/25 (8%) showed an increase compared with baseline; clinical improvement was observed irrespective of IgE change* (*p* < 0.001). Total IgE levels decreased by a mean of 1095.1 ± 2169.0 (95% CI 199.8–1990.5) in all patients after OMZ treatment (*p* < 0.001) (Figure 1). Table 2 presents the clinical parameters of the patients before and after treatment.

Factors associated with the mean decrease in BPDAI score after treatment were evaluated. A weak positive correlation was observed between the decrease in BPDAI score and the maximum systemic corticosteroid dose used before treatment (r = 0.400; *p* < 0.05). A very strong positive correlation was found between baseline BPDAI score and the magnitude of its reduction (r = 0.995; *p* < 0.01). In exploratory analyses, no significant correlation was observed between the corticosteroid dose used during treatment and the decrease in BPDAI (*p* > 0.05), whereas the reduction strongly correlated with baseline BPDAI (r = 0.995; *p* < 0.01). No significant correlations were detected between the decrease in BPDAI score and pretreatment peripheral eosinophil count, total IgE levels, pruritus VAS, corticosteroid dose during treatment, age, or BMI (*p* > 0.05).

Evaluation of the factors associated with the decrease in systemic corticosteroid levels after treatment was performed. There was a weak positive correlation between the decrease in corticosteroid dose and pretreatment peripheral blood eosinophil level (r = 0.482; *p* < 0.05). There was a high positive correlation with the maximum corticosteroid dose before treatment (r = 0.838; *p* < 0.01). No significant correlation was observed between the decrease in corticosteroid dose and sociodemographic and clinical characteristics of the patients (*p* = 0.05).

A significant correlation was observed between the mean decrease in BPDAI score and blood hypertension (130.0 ± 41.7; 91.0 ± 48.1 *p*: 0.03). However, no statistically significant difference was observed between gender, other comorbidities, and clinical characteristics, and the mean decrease in BPDAI score (*p* = 0.05).

Treatment response onset time (weeks) and associated factors were evaluated. A statistically significant difference was observed between treatment response onset time (weeks) and hyperlipidemia (8.1 ± 3.7, 5.1 ± 2.5; *p* = 0.04). Regression analysis showed that hyperlipidemia was not an independent risk factor affecting treatment response at the onset time.

Systemic corticosteroid dose reduction time (weeks) and associated factors were evaluated by treatment. There was no significant correlation with pretreatment clinical parameters (blood eosinophil level, blood total IgE level, pretreatment pruritus VAS, corticosteroid dose used during treatment, and BMI). There was a moderate negative correlation between the age of the patients and the corticosteroid dose reduction time (weeks) (r = −0.424; *p* < 0.05). Regression analysis showed that a one-unit increase in age was an independent risk factor decreasing the corticosteroid dose reduction time (OR: 0.069; 95% CI: 0.125–0.013).

## 4. Discussion

Bullous pemphigoid (BP) is the most common autoimmune blistering disorder in older adults, with annual incidence estimates ranging from 2.5 to 42.8 cases per million worldwide and characterized by tense subepidermal blisters [10]. While high baseline blood total IgE levels have been associated with better responses in some studies, our findings suggest that *patients without significant total IgE reduction may still benefit clinically from OMZ, as shown by two cases in this study*. Although BP is seldom life-threatening, its relentless pruritus can severely impair sleep and health-related quality of life (HRQoL) [11]. Systemic corticosteroids remain the therapeutic cornerstone; however, prolonged use doubles the risk of serious infections and may severely affect organ systems in this vulnerable population. The goal of treatment is to use the lowest possible dose of corticosteroid therapy as soon as possible [12]. These facts have prompted the search for safer yet effective alternatives. Omalizumab (OMZ), an anti-IgE monoclonal antibody, has recently shown encouraging outcomes, achieving complete remission in up to 77% of steroid-refractory BP cases in the largest multicenter series to date [7]. This retrospective study provides real-world data from a tertiary dermatology center on the use of OMZ in BP patients with severe, sleep-disrupting pruritus who were either intolerant of systemic corticosteroids or had contraindications to their continued use. In this cohort, triggers for OMZ use were predominantly steroid-refractory disease and persistent pruritus compromising sleep/HRQoL, with frailty, contraindications to immunosuppressants, and partial corticosteroid response based on clinician judgment also contributing. In our cohort, systemic corticosteroids were tapered at approximately four-week intervals only after predefined improvement criteria were met to minimize confounding from corticosteroid effects and to address adverse effects or intolerance. This timing was chosen to reduce the likelihood that early clinical improvement was attributable solely to corticosteroids, particularly as all patients started OMZ due to corticosteroid-refractory disease or intolerance. This operational difference—tapering at ~4 weeks versus a median documented response at 6.4 weeks—indicates that early improvements may reflect combined corticosteroid and omalizumab effects; accordingly, these findings should be interpreted as hypothesis-generating rather than proof of causality.

In the largest multicenter study to date (*n* = 100), Chebani et al. achieved a 77% complete remission rate with OMZ; however, only 11.7% of those remissions were obtained off systemic therapy, and the investigators did not capture quantitative itch data such as pruritus VAS [7]. A subsequent systematic review pooling 53 published cases confirmed respectable efficacy (55% complete and 32% partial responses), yet fewer than one-third of patients discontinued concomitant immunosuppressants, and pruritus scores were again largely absent [11]. Turkish experience has so far been confined to single-center reports from İstanbul (15 patients) [5] and Ankara (11 patients) [12], both of which concentrated mainly on lesion clearance and provided minimal itch quantification. Against this backdrop, our 25-patient cohort—the largest to date from Türkiye—evaluates OMZ initiated as an add-on to systemic corticosteroids in a steroid-sparing context. Within a pre-specified 12-week window, we observed a mean 105-point reduction in BPDAI and a 6.2-point decrease in pruritus VAS. Systemic corticosteroids were tapered per predefined criteria, and by follow-up, many patients were maintained on OMZ alone. Given the uncontrolled add-on design, these findings are hypothesis-generating rather than definitive proof of efficacy. From a practical standpoint, in elderly BP with steroid-refractory or intolerant disease and sleep-disrupting pruritus, OMZ given at 300 mg/4 weeks as an add-on may be considered, with reassessment at ~4 weeks and taper steps pre-specified to minimize confounding. Prospective algorithms comparing OMZ with dupilumab and standardized taper protocols are warranted.

At the pre-OMZ baseline (immediately before first injection), total IgE was above the laboratory upper limit of normal in all 25/25 patients; by week 12, it declined in 23/25 (92%), whereas 2/25 showed an increase yet nonetheless *improved clinically*; taken together, IgE dynamics broadly paralleled the mean 6.2-point reduction in VAS–pruritus and the ~105-point fall in BPDAI, suggesting that early IgE decline often mirrors—but is not requisite for—symptom relief and objective disease control.

Notably, clinical benefit was also observed in these two patients, indicating that omalizumab’s effects may extend beyond total IgE reduction. By contrast, a French multicenter series of 100 refractory patients reported excellent lesion clearance but found that blood total IgE concentrations did not predict response and remained elevated in most responders, while quantitative itch data were not collected [7]. For this reason, we believe that our data add real-world evidence on itch quantification and IgE dynamics in an add-on, steroid-sparing context. This observation aligns with previous reports indicating that OMZ efficacy is not strictly dependent on total IgE decline, supporting the concept of additional immunomodulatory mechanisms beyond IgE neutralization. Likewise, a 53-patient systematic review pooling published experience was unable to analyze IgE dynamics because fewer than one-third of the included reports provided serial values or a validated pruritus scale. The only study that detailed IgE kinetics—in a smaller cohort—reported a ~25% decline (1102 ± 835 to 835 ± 614 IU/mL) over several months [5]. In our add-on, steroid-sparing cohort, median total IgE showed a significant decrease at 12 weeks, and 92% of patients showed a decline. While design and follow-up differences preclude direct comparison, the short-term reduction in our series appears more pronounced. Taken together, these comparisons highlight two strengths of the present work. First, we show that omalizumab may rapidly reduce total IgE while delivering parallel improvements in BPDAI and in quality-of-life–impairing pruritus, as captured by pruritus VAS. Second, we provide the first medium-sized, systematically monitored dataset from Türkiye linking dynamic IgE changes with both itch relief and steroid-sparing efficacy. IgE’s role in BP pathogenesis is well established, with over 70% of BP patients having elevated IgE levels, which provides a biologic rationale for evaluating anti-IgE therapy such as OMZ in selected patients. From 2009 to 2022, OMZ has shown significant efficacy in most patients, though a small subset experience treatment failure or adverse effects, such as thrombocytopenia and BP flare-ups. A clear dose–response relationship has not been established; however, some series report complete responses after more than five doses. Achieving a complete response rate of over 70% typically requires more than five doses of OMZ [5]. Ferhatoğlu et al. showed that total IgE falls in tandem with clinical benefit: their 15-patient series recorded a mean 268 IU/mL drop in IgE and had two-thirds of patients were steroid-free by week 8. The short-term decline in total IgE observed here is unlikely to be attributable to concomitant systemic corticosteroids; indeed, multiple reports describe transient rises in serum IgE during the first 1–2 weeks of systemic corticosteroid therapy [13,14,15,16]. Conversely, the pattern is biologically consistent with omalizumab’s mechanism—rapid neutralization of free IgE followed by down-regulation of FcεRI on effector cells [4,7,17,18]. Taken together, our data are concordant with contemporary reports and add real-world value; in patients with elevated total IgE and pruritus-predominant BP, earlier initiation of OMZ may help reduce itch burden and thereby improve HRQoL.

The above-mentioned study by Ferhatoğlu et al. also revealed no significant decrease in eosinophil levels after treatment [5]. Alexandre et al. echoed the rapid response profile, reporting visible lesion healing within one month and an 85% complete remission rate when OMZ was added for pemphigoid at therapeutic impasse [7,8]. In our cohort, both blood total IgE and eosinophil counts declined significantly, and these laboratory shifts paralleled marked improvements in BPDAI, pruritus VAS, and early corticosteroid independence. Because our study is retrospective and lacks laboratory checkpoints at months 1, 3, and 6, prospective work with scheduled sampling is needed to confirm whether early total IgE and eosinophil reductions, in addition to clinical well-being, reliably forecast durable remission. These findings, together with the observation that two patients improved clinically despite rising total IgE, support additional mechanisms beyond IgE neutralization—such as rapid down-regulation of FcεRI in basophils/mast cells and reduced eosinophil trafficking—which may be particularly relevant in pruritus-dominant BP.

In the present study, treatment led to a significant reduction in systemic corticosteroid use, which is particularly important given the potential side effects of prolonged corticosteroid therapy. This decrease may highlight omalizumab’s potential to manage the disease effectively while reducing the need for corticosteroids, offering a safer and possibly more sustainable treatment approach for patients. Overall, these results strongly support the therapeutic value of OMZ in reducing disease activity, blood eosinophil count, and corticosteroid dependence, making it a promising treatment option. Chebani et al. reported the largest series to date of BP treated with OMZ, confirming its effectiveness and safety for this indication [1,7]. Importantly, their findings suggest that baseline serum levels of anti-BP180-NC16A IgE could serve as a predictor of treatment response, offering potential for more personalized and targeted therapy. This adds valuable insight into optimizing omalizumab use in BP patients. Seyed Jafari’s observations suggest that OMZ offers a promising therapeutic option for managing BP patients [2,6]. Its effectiveness may be attributed to its ability to reduce FcεRI+ and IgE+ basophils, as well as intradermal cells, which are key players in the inflammatory process. This mechanism of action could help target the underlying immune response, making OMZ a valuable treatment for BP.

In this current study, systemic corticosteroid dose reduction time had no significant correlation with pretreatment clinical parameters. However, age showed a moderate negative correlation, with older patients experiencing a shorter reduction time. This may be due to reasons unrelated to OMZ, such as physicians being more sensitive to possible side effects, but further research is needed. Regression analysis identified age as an independent risk factor for faster corticosteroid dose reduction, with no significant links to other sociodemographic or clinical factors. While pretreatment clinical parameters did not significantly correlate with systemic corticosteroid reduction time, age was found to be a key factor, with older patients experiencing faster reduction. Our data suggest potential steroid-sparing signals of OMZ, but randomized studies are needed to confirm effectiveness and safety. This makes OMZ a promising alternative, especially for patients who are either unable to tolerate immunosuppressive therapies or have not responded to conventional treatments. Despite these encouraging findings, randomized controlled trials are essential to provide stronger evidence and further validate the efficacy and safety of OMZ in the treatment of BP [6]. OMZ appears promising as an effective therapeutic option for BP, as it led to clinical improvement in many patients, with nearly half achieving a complete response. Importantly, it was well tolerated with no significant side effects, making it especially beneficial for elderly BP patients who are more vulnerable to treatment-related complications. Baseline serum total IgE and eosinophil levels did not predict treatment response, indicating these values should not guide patient selection for OMZ. All patients receive 300 mg every four weeks, a dosing regimen that has been approved for chronic spontaneous urticaria. This regimen is well tolerated and effective, making it a reasonable starting point for future BP studies.

The recent literature has increasingly explored the role of biologic agents in the management of BP, particularly in refractory cases. In line with our findings, a recent systematic review by Granados-Betancort et al. comprehensively evaluated the efficacy and safety of OMZ and dupilumab for BP, summarizing evidence from case reports, case series, and small clinical studies. Their analysis supports the growing body of evidence indicating that OMZ can achieve favorable clinical outcomes with an acceptable safety profile, especially in patients who are unresponsive or intolerant to conventional immunosuppressive therapies [17]. The present study adds to this body of work by providing further clinical evidence on the potential benefits of OMZ, thereby reinforcing the rationale for its consideration as a therapeutic option in BP.

### 4.1. Strengths

This study comprehensively evaluates multiple clinically relevant disease markers, such as corticosteroid dose reduction, blood eosinophil count, pruritus severity, and BPDAI scores, providing a multidimensional assessment of treatment efficacy. It underscores omalizumab’s potential to reduce corticosteroid dependency and thereby minimize corticosteroid-related adverse effects. Moreover, by focusing on a refractory patient population, the findings contribute valuable real-world evidence supporting OMZ as an effective therapeutic alternative for difficult-to-treat bullous pemphigoid cases.

### 4.2. Limitations

The relatively small sample size and the absence of a control group limit the generalizability and the strength of causal inferences in this study. Without a comparator arm, it is challenging to attribute the observed clinical improvements exclusively to omalizumab, as confounding factors cannot be fully excluded. In particular, the add-on design with a corticosteroid taper introduces time-varying confounding, regression-to-the-mean, and potential indication bias, which preclude definitive causal attribution despite steps taken to minimize confounding. Furthermore, the retrospective design imposes inherent limitations related to potential selection bias and missing data. Finally, long-term efficacy and safety outcomes remain unaddressed, underscoring the need for prospective controlled studies to validate these findings.

The choice of OMZ over other systemic immunosuppressants was influenced by advanced patient age, comorbidities, contraindications to agents like azathioprine and mycophenolate, and the predominance of severe pruritus, supporting a favorable safety profile in this setting.

## 5. Conclusions

This study highlights the significant efficacy of OMZ in reducing BPDAI scores and improving key disease markers, including corticosteroid dose, pruritus VAS score, total IgE levels, and blood eosinophil count. The findings indicate that the reduction in BPDAI score is positively correlated with treatment response onset and the maximum corticosteroid dose used before treatment. Additionally, older age was identified as an independent risk factor for a shorter corticosteroid dose reduction time. Overall, our study provides hypothesis-generating real-world data suggesting that omalizumab may offer rapid itch relief and potential steroid-sparing benefit in selected patients. However, further research is needed to identify factors predicting treatment response.

## Figures and Tables

**Table 1:** Sociodemographic and clinical characteristics of patients.

Characteristics	
**Age** (year)	71.3 ± 8.4
**Gender** (Female)	19 (76.0%)
**Height** (cm)	164.2 ± 9.1/160.0 (157–174)
**Weight** (kg)	76.8 ± 7.3/78.0 (75–81.5)
**BMI** (kg/m^2^)	28.7 ± 3.7
**Smoking**	3 (12.0%)
**Comorbidity**	
*Diabetes mellitus*	14 (56.0%)
*Hypertension*	16 (64.0%)
*Hyperlipidemia*	14 (56.0%)
*Malignancy*	0 (0%)
**Disease duration** (months)	4.4 ± 2.5
**Lesion Areas** (%)	
*Trunk*	24 (96.0%)
*Genital Area*	10 (40.0%)
*Extremity*	23 (92.0%)
*Oral Mucosa*	13 (52.0%)
*Scalp*	9 (36.0%)
**BP180**	25 (100.0%)
**BP230**	25 (100.0%)
**Time to onset of response to treatment** (weeks)	6.4 ± 3.3
**Duration of corticosteroid decrease after OMZ treatment** (weeks)	3.8 ± 1.2/4.0 (3–4)
**Minimum corticosteroid dose given in OMZ treatment** (mg)	2.0 ± 2.5/0 (0–5)
Mean ± standard deviation/median (25.–75. percentile) *n* (%)	

**Table 2:** Evaluation of clinical parameters of patients before and after omalizumab (OMZ) treatment.

Parameters	OMZ Treatment	Mean ± Standard Deviation Median (25th–75th Percentile)	*p*-Value
**Eosinophil (cells/μL)**	Before	0.7 ± 0.4/ 0.8 (0.3–1.1)	<0.001
	After	0.2 ± 0.1/ 0.1 (0.01–0.2)	
**Total IgE (IU/mL)**	Before	1946.7 ± 2764.1/ 729.0 (279–2814)	<0.001
	After	851.6 ± 1205.8/ 315.0 (63–1179)	
**BPDAI Score**	Before	116.2 ± 53.3/ 103.0 (72–158)	<0.001
	After	11.1 ± 6.2/ 10.0 (8–13)	
**Pruritus–VAS Score**	Before	7.9 ± 1.5/ 8.0 (7.0–9.0)	<0.001
	After	1.7 ± 1.1/ 2.0 (1.0–2.5)	
**Systemic Corticosteroid Dose**	Before	43.2 ± 14.8/ 40.0 (32–60)	<0.001
	After	6.2 ± 8.9/ 0 (0–18)	

Wilcoxon rank test; *p* < 0.05, statistically significant; BPDAI, bullous pemphigoid disease area index.

## Data Availability

The data is available upon request.

## References

[1] Vassallo C., Somenzi A., De Amici M., Barruscotti S., Brazzelli V. (2022). Omalizumab as a corticosteroid-sparing agent in the treatment of bullous pemphigoid. Dermatol. Ther..

[2] Ling X., Shou X., Lou Y., Ling J., Zhang M., Yu T., Gu W. (2023). Research progress of omalizumab in the treatment of bullous pemphigoid. J. Dermatol..

[3] Zeng F.A.P., Wilson A., Sheriff T., Murrell D.F. (2022). Side effects of steroid-sparing agents in patients with bullous pemphigoid and pemphigus: A systematic review. JAAD Int..

[4] Poddighe D., Vangelista L. (2020). Effects of omalizumab on basophils: Potential biomarkers in asthma and chronic spontaneous urticaria. Cell Immunol..

[5] Ferhatoglu Z.A., Yucesoy S.N., Ak T., Demir Y. (2024). Omalizumab in the treatment of bullous pemphigoid: A single-center series of 15 cases. Medicine.

[6] Morteza Seyed Jafari S., Gadaldi K., Feldmeyer L., Yawalkar N., Borradori L., Schlapbach C. (2019). Effects of omalizumab on FcεRI and IgE expression in lesional skin of bullous pemphigoid. Front. Immunol..

[7] Chebani R., Lombart F., Chaby G., Dadban A., Debarbieux S., Viguier M.A., Ingen-Housz-Oro S., Pham-Ledard A., Bedane C.R., Picard-Dahan C. (2024). Omalizumab in the treatment of bullous pemphigoid resistant to first-line therapy: A French national multicentre retrospective study of 100 patients. Br. J. Dermatol..

[8] Alexandre M., Bohelay G., Gille T., Le Roux-Villet C., Soued I., Morin F., Caux F., Grootenboer-Mignot S., Prost-Squarcioni C. (2022). Rapid Disease Control in First-Line Therapy-Resistant Mucous Membrane Pemphigoid and Bullous Pemphigoid with Omalizumab as Add-On Therapy: A Case Series of 13 Patients. Front. Immunol..

[9] Rosi-Schumacher M., Baker J., Waris J., Seiffert-Sinha K., Sinha A.A. (2023). Worldwide epidemiologic factors in pemphigus vulgaris and bullous pemphigoid. Front. Immunol..

[10] Miyamoto D., Santi C.G., Aoki V., Maruta C.W. (2019). Bullous pemphigoid. An. Bras. Dermatol..

[11] D’Aguanno K., Gabrielli S., Ouchene L., Muntyanu A., Ben-Shoshan M., Zhang X., Iannattone L., Netchiporouk E. (2022). Omalizumab for the Treatment of Bullous Pemphigoid: A Systematic Review of Efficacy and Safety. J. Cutan. Med. Surg..

[12] Uysal P.I., Yalcin B., Oktem A. (2017). Our clinical experience with the use of omalizumab in the treatment of bullous pemphigoid. Turkderm-Turk. Arch. Dermatol. Venereol..

[13] Zieg G., Lack G., Harbeck R.J., Gelfand E.W., Leung D.Y. (1994). In vivo effects of glucocorticoids on IgE production. J. Allergy Clin. Immunol..

[14] Barnes P.J. (2001). Corticosteroids, IgE, and atopy. J. Clin. Investig..

[15] Lim J., Lin E.V., Hong J.Y., Vaidyanathan B., Erickson S.A., Annicelli C., Medzhitov R. (2022). Induction of natural IgE by glucocorticoids. J. Exp. Med..

[16] Salvi S.S., Babu K.S., Holgate S.T. (2000). Glucocorticoids enhance IgE synthesis. Are we heading towards new paradigms?. Clin. Exp. Allergy.

[17] Granados-Betancort E., Sánchez-Díaz M., Muñoz-Barba D., Arias-Santiago S. (2024). Omalizumab and Dupilumab for the Treatment of Bullous Pemphigoid: A Systematic Review. J. Clin. Med..

[18] Maurer M., Rosén K., Hsieh H.J., Saini S., Grattan C., Gimenéz-Arnau A., Agarwal S., Doyle R., Canvin J., Kaplan A. (2013). Omalizumab for the Treatment of Chronic Idiopathic or Spontaneous Urticaria. N. Engl. J. Med..

